# Meta-transcriptomics for the diversity of tick-borne virus in Nujiang, Yunnan Province

**DOI:** 10.3389/fcimb.2023.1283019

**Published:** 2023-12-15

**Authors:** Juan Wang, Jing Wang, Guopeng Kuang, Weichen Wu, Lifen Yang, Weihong Yang, Hong Pan, Xi Han, Tian Yang, Mang Shi, Yun Feng

**Affiliations:** ^1^ Yunnan Provincial Key Laboratory for Zoonosis Control and Prevention, Yunnan Institute of Endemic Disease Control and Prevention, Dali, China; ^2^ State Key Laboratory for Biocontrol, School of Medicine, Shenzhen Key Laboratory for Systems Medicine in Inflammatory Diseases, Sun Yat-sen University, Shenzhen, China; ^3^ School of Public Health, Dali University, Dali, China; ^4^ State Key Laboratory of Remote Sensing Science, Center for Global Change and Public Health, Faculty of Geographical Science, Beijing Normal University, Beijing, China

**Keywords:** tick, tick-borne virus, arboviruses, meta-transcriptomic, Yunnan

## Abstract

Ticks, an arthropod known for transmitting various pathogens such as viruses, bacteria, and fungi, pose a perpetual public health concern. A total of 2,570 ticks collected from Nujiang Prefecture in Yunnan Province between 2017 and 2022 were included in the study. Through the meta-transcriptomic sequencing of four locally distributed tick species, we identified 13 RNA viruses belonging to eight viral families, namely, *Phenuiviridae*, *Nairoviridae*, *Peribunyaviridae*, *Flaviviridae*, *Chuviridae*, *Rhabdoviridae*, *Orthomyxoviridae*, and *Totiviridae*. The most prevalent viruses were members of the order *Bunyavirales*, including three of *Phenuiviridae*, two were classified as *Peribunyaviridae*, and one was associated with *Nairoviridae*. However, whether they pose a threat to human health still remains unclear. Indeed, this study revealed the genetic diversity of tick species and tick-borne viruses in Nujiang Prefecture based on COI gene and tick-borne virus research. These data clarified the genetic evolution of some RNA viruses and furthered our understanding of the distribution pattern of tick-borne pathogens, highlighting the importance and necessity of monitoring tick-borne pathogens.

## 1 Introduction

Ticks are distributed worldwide and parasitize various host groups including mammals, amphibians, and birds. Currently, at least 125 species have been reported in China, including 111 species belonging to seven genera of hard ticks and 14 species from two soft tick genera (Zhang et al., 2019). They can infect viruses, bacteria, protozoa, and other microbes from different hosts and transmit them between hosts (Zhang et al., 2019). Tick-borne viruses pose a significant health threat to human and animal populations. With the application of high-throughput sequencing technology, many novel tick-borne bacteria and viruses have been identified in various ticks around the world, including countries like Korea, Mongolia, Nigeria, and China (Kamani et al., 2019; Altantogtokh et al., 2022; Lee et al., 2023; Yang et al., 2023). Researchers detected numerous arboviral viruses by next-generation sequencing (NGS) technology in 640 ticks collected in eight provinces in central and western China, including 13 novel viruses (Yang et al., 2023). Indeed, the tick-borne viruses comprise two orders, nine families, and at least 12 genera, alongside some that are still unclassified (Shi et al., 2018). NGS has invariably fast-tracked the discovery of pathogenic viruses, enriching our understanding of the composition and evolution of tick-borne viruses.

Currently, numerous specific tick-borne diseases of humans and mammals have been described. For instance, the Severe Fever with Thrombocytopenia Syndrome Virus (SFTSV), first isolated from febrile patients’ blood in 2010, can cause fever, thrombocytopenia, leukocytopenia, and multiorgan organ dysfunction in humans (Yu et al., 2011). Additionally, the Alongshan virus, which was detected in the blood of febrile patients, has been shown to infect humans (Wang et al., 2019). Other tick-borne viruses such as Beiji Nairovirus (BJNV), Jingmen tick virus (JMTV), and Tacheng tick virus 2 (TcTV-2) have similarly been associated with febrile illnesses in humans (Jia et al., 2019; Dong et al., 2021; Wang et al., 2021b). The upward trend in emerging tick-borne viruses indicates the increasing threat these pose globally, thereby underscoring the challenges faced in preventing and controlling emerging infectious diseases.

Nujiang Prefecture, situated in northwest Yunnan Province, boasts a unique vertical natural landscape characterized by towering mountains and intricate canyons. Benefiting from its unique stereoclimatic conditions, the region formed diverse ecosystems that provide suitable habitats for various arthropods and other biological resources. In recent years, tick-borne pathogens have also been detected in Yunnan Province. The Dabieshan tick virus (DTV) was first identified in *Rhipicephalus microplus* within this province, suggesting that DTV may be widely prevalent in southwestern China (Wang et al., 2021a). In Luxi County, Yunnan Province, the genomic sequences of a novel Jingmen tick virus (JMTV) were discovered in the *Rhipicephalus microplus* in 2017 (Shi et al., 2021). When researchers conducted a screening of female ticks, tick eggs, and larvae of *Haemaphysalis longicornis* in Yunnan Province, they confirmed that the JMTV RNA positivity rate is higher than 8% and demonstrated a substantial degree of evolutionary conservatism (Wu et al., 2023). Despite this, there still need to be studies investigating the viral spectrum of ticks in Nujiang. In this study, we identified 13 RNA viruses from eight families by meta-transcriptome analysis, even though some were potentially associated with severe human diseases.

## 2 Materials and methods

### 2.1 Sample collection and sample processing

This study was carried out in Nujiang, located at the northwestern border of Yunnan Province. Marked by its border with Tibet to the north and Myanmar to the west, Nujiang’s favorable climate and rich species resources suit diverse tick-borne species. In order to investigate the distribution of pathogen-carrying ticks, samples were collected in all four counties of Nujiang, namely, Lushui (LS), Fugong (FG), Lanping (LP), and Gongshan (GS). Ticks were collected from stocked cattle and sheep using forceps between April and October each year from 2017 to 2022 and then grouped into 15-ml tubes according to their collection location. These samples were transported to the laboratory in liquid nitrogen and stored at −80°C until further processing. Ticks were preliminary identified and grouped by morphological traits, and species identification was performed after the sample processing and sequencing based on the analysis of the cytochrome C oxidase subunit I (COI) gene by meta-transcriptomic sequencing (Hebert et al., 2003).

### 2.2 RNA extraction, library construction, and sequencing

The samples were divided into 30 groups according to time, location, and morphology information. These ticks were then homogenized in the frozen mortar with 500 µl MEM and then centrifuged at 9,000 rpm for 20 min at 4°C. The supernatant was collected and stored at −80°C. Total RNA from each pool was extracted and purified using the RNeasy Plus Universal Mini Kit (QIAGEN, Germany) following the manufacturer’s instructions. Meta-transcriptome library construction and ribosomal RNA depletion were carried out using the Zymo-Seq RiboFree Total RNA Library Kit (Zymo Research, USA). Each RNA library was subsequently subjected to paired-end (150 bp) sequencing using the Illumina NovaSeq 6000 platform.

### 2.3 Virus discovery and genome annotation

High-quality reads were obtained by removing Adaptor sequences and low-quality reads from the raw sequencing reads. The clean reads were then assembled *de novo* into contigs using MEGAHIT (version 1.2.8) (Li et al., 2015b). The assembled contigs were then compared against the non-redundant protein (nr) database from GenBank by using Diamond blastx (version 0.9.25) (Buchfink et al., 2021), and the E value threshold was set at 1E-5 to assure high sensitivity with a low false-positive rate. Viral contigs were acquired based on the taxonomic information from the blast hits and then merged into longer genomes using the SeqMan program in the Lasergene package version 7.1 (DNAstar) (Clewley, 1995). Reads were mapped back to the viral genome to eliminate misassembly using Bowtie2 (version 2.3.5.1), and the final viral genome was examined using Geneious Prime (version v.9.1.5) (Kearse et al., 2012; Deorowicz et al., 2019). To identify novel virus species, we evaluated and compared the RdRp protein (RNA viridae), NSP1 protein (*Flaviviridae*), and PB1 protein (*Totiviridae*) of viruses with the threshold of the consensus <90% amino acid identity. The sequences were also compared with the sequence of the closest related viruses and confirmed by phylogenetic analysis. We used “Yunnan” as a taxonomic marker to distinguish them from other viral strains.

### 2.4 Viral abundance estimation and phylogenetic analysis

To estimate the abundance of each virus in each library, we initially removed clean reads associated with rRNAs from the ribosomal RNA database sourced from the SILVA website (https://www.arb-silva.de/) using Bowtie2 (Langmead and Salzberg, 2012). Upon this, we estimated the abundance of each virus based on the sequence reads mapped to the number of reads per million (RPM) in each library on the viral reference genome. Representative amino acid sequences with high similarity to the obtained viral sequences were downloaded from NCBI as reference sequences and compared with viral sequences obtained from this study using MAFFT (version 7) (Katoh and Standley, 2013). The ambiguous alignment region was removed using trimal (version 1.2) (Capella-Gutiérrez et al., 2009) and the software Mega (version 5.2) (Tamura et al., 2011). Phylogenetic trees were inferred using the maximum likelihood method in PhyML (version 3.1) with the LG amino acid substitution model and SPR structural optimization algorithm (Guindon et al., 2010).

## 3 Results

### 3.1 Samples and identification

**Figure 1:**
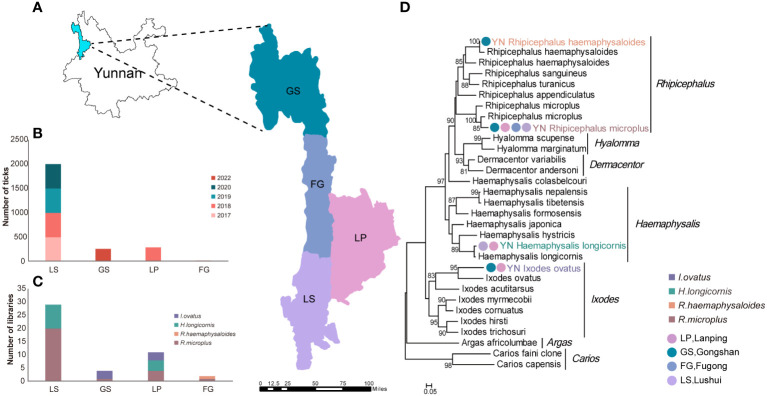
Sampling locations and tick species identification in Nujiang, Yunnan. **(A)** Sample collection sites in this study. **(B)** The number of survey specimens brought into the library at each sampling site. **(C)** Number of libraries of each tick species per sampling site. **(D)** Ticks species identification based on phylogenetic analysis of the COI Gene in Nujiang.

After screening out ticks with incomplete limbs, a total of 2,570 ticks collected in Nujiang were included in this study, including 2,000 ticks from Lushui between 2017 and 2020, 290 ticks from Lanping in 2018, 20 ticks from Fugong in 2022, and 260 ticks from Gongshan in 2022 (**Figures 1A, B**, **Table S1**). The samples were grouped into 30 pools based on the morphology, the collection time, and locations. The analysis of COI genes of each pool identified four tick species belonging to three genera in Nujiang, including two species from *Rhipicephalus* (*R. microplus* and *R. haemaphysaloides*), one from *Haemaphysalis* (*H. longicornis*), and one from *Ixodes* (*I. ovatus*) (**Figure 1D**, **Table S2**). Among these, *R. microplus* was found to be widely distributed in Nujiang, being detected in 26 libraries at four sampling sites, then *H. longicornis* followed, found across 13 libraries in LS and LP, and *I. ovatus* was discovered in LP and GS; in contrast, *R. haemaphysaloides* showed a limited distribution which was found only in FG (**Figure 1C**, **Table S2**).

### 3.2 Viral diversity and abundance of ticks in Nujiang

To investigate the virome carried by ticks, we performed high-throughput sequencing on 30 meta-transcriptome libraries at a depth of 10 G and eventually obtained 74.4 GB of zipped data. After the quality control process, we obtained 2,018,111,978 reads, and assembled *de novo* into 1,230,648 contigs. 3,397 contigs were identified as viral sequences (**Table S2**). Based on blast alignment and the RdRp homology, we identified 13 virus species from eight viral families, including single-stranded positive-sense RNA viruses (family: *Flaviviridae*); negative-stranded RNA viruses (families: *Phenuiviridae*, *Peribunyaviridae*, *Nairoviridae*, *Chuviridae*, *Rhabdoviridae*, *Orthomyxoviridae*), and a double-stranded RNA virus (family: *Totiviridae*) (**Table 1**). Of these, eight viruses displayed high amino acid similarity (>90%) to previously identified tick-associated viruses, which were tentatively named Yunnan phlebovirus 1 (YPV1), Yunnan phlebovirus 2 (YPV2), Yunnan flavi-like virus (YFV), Yunnan rhabdo-like virus 1 (YRV1), Yunnan rhabdo-like virus 2 (YRV2), Yunnan mivirus (YMV), Yunnan orthomy-like virus (YOV), and Jingmen tick virus (JMTV) (**Table 1**). Some are yet to be categorized by the International Committee on Taxonomy of Viruses (ICTV). The remaining five species were thought to be novel viral species, four significantly divergent from previously identified viruses, namely Yunnan nairo-like virus (YNV), Yunnan ixovirus (YIV), Yunnan peribunya-like virus 1 (YPBV1), Yunnan peribunya-like virus 2 (YPBV2), and Yunnan toti-like virus (YTV) (**Table 1**). No DNA viruses were found in this study.

**Table 1:** Thirteen tick-borne viruses identified in this study.

Order	Family	Virus name	Genome segment	Length	Blastx hits on known viruses	GenBank accession number of genetically closest virus
Bunyavirales	Nairoviridae	Yunnan nairo-like virus	L	15,007	Sichuan tick nairovirus (65.31%)	USZ80660.1
			S	3,618	Sichuan tick nairovirus (54.91%)	USZ80657
	Phenuiviridae	Yunnan phlebovirus virus 1	L	6,442	Mukawa virus (91.80%)	WAK75785.1
			M	3,330	Mukawa virus (85.26%)	YP_009666331.1
			NP	748	Mukawa virus (85.54%)	UUT07166.1
		Yunnan phlebovirus virus 2	L	6,496	Lihan tick virus (99.81%)	WAK76765.1
			S	1,778	*R.* associated phlebovirus 1 (99.79%)	QCB64645.1
		Yunnan ixovirus	L	6,740	Blacklegged tick phlebovirus 2 (61.23%)	AII01803.1
			S	2,502	Sara tick phlebovirus (49.32%%)	QPD01620.1
	Peribunyaviridae	Yunnan peribunya-like virus 1	L	9,205	Bronnoya virus (62.71%)	WCD38987.1
			M	4,274	Bronnoya virus (53.22%)	WCD38995.1
		Yunnan peribunya-like virus 2	L	9,106	Yushu tick virus 1 (79.73%)	UYL95520.1
			M	2,834	Fuhai tick bunyavirus (85.76%)	UXL90890.1
Amarillovirales	Flaviviridae	Jingmen tick virus	NSP1	3,121	Jingmen tick virus (99.12%)	USE57273.1
			VPI	2,762	Jingmen tick virus (97.35%)	USE57274.1
			NSP2	2,850	Jingmen tick virus (99.12%)	YP_009030000.1
			VP2/3	2,802	Jingmen tick virus (98.70%%)	WJJ44609.1
		Yunnan flavi-like virus	Partial genome	5,729	Flaviviridae sp. (95.54%)	UGM45976.1
Mononegavirales	Rhabdoviridae	Yunnan rhabdo-like virus 1	Nearly complete genome	10,057	*R.* associated rhabdo-like virus (99.41%)	WAK76863.1
		Yunnan rhabdo-like virus 2	Nearly complete genome	11,560	Rhabdoviridae sp. (99.68%)	WAK76971.1
Ghabrivirales	Totiviridae	Yunnan toti-like virus	Partial genome	8,062	Totiviridae sp. (74.48%)	WAK77279.1
Jingchuvirales	Chuviridae	Yunnan mivirus	Nearly complete genome	11,553	Wuhan mivirus (99.73%)	WAS28395.1
Articulavirales	Orthomyxoviridae	Yunnan orthomy-like virus	HA	1,811	Quaranjavirus sp. (90.23%)	URY50687.1
			NP	1,625	Guangdong tick quaranjavirus (87.98%)	WAA68656.1
			PA	2,389	Guangdong tick quaranjavirus (91.51%)	WAA68654.1
			PB1	2,362	Orthomyxoviridae sp. (96.50%)	UUT42615.1
			PB2	2,422	Guangdong tick quaranjavirus (92.76%)	WAA68653.1

R, Rhipicephalus.

**Figure 2:**
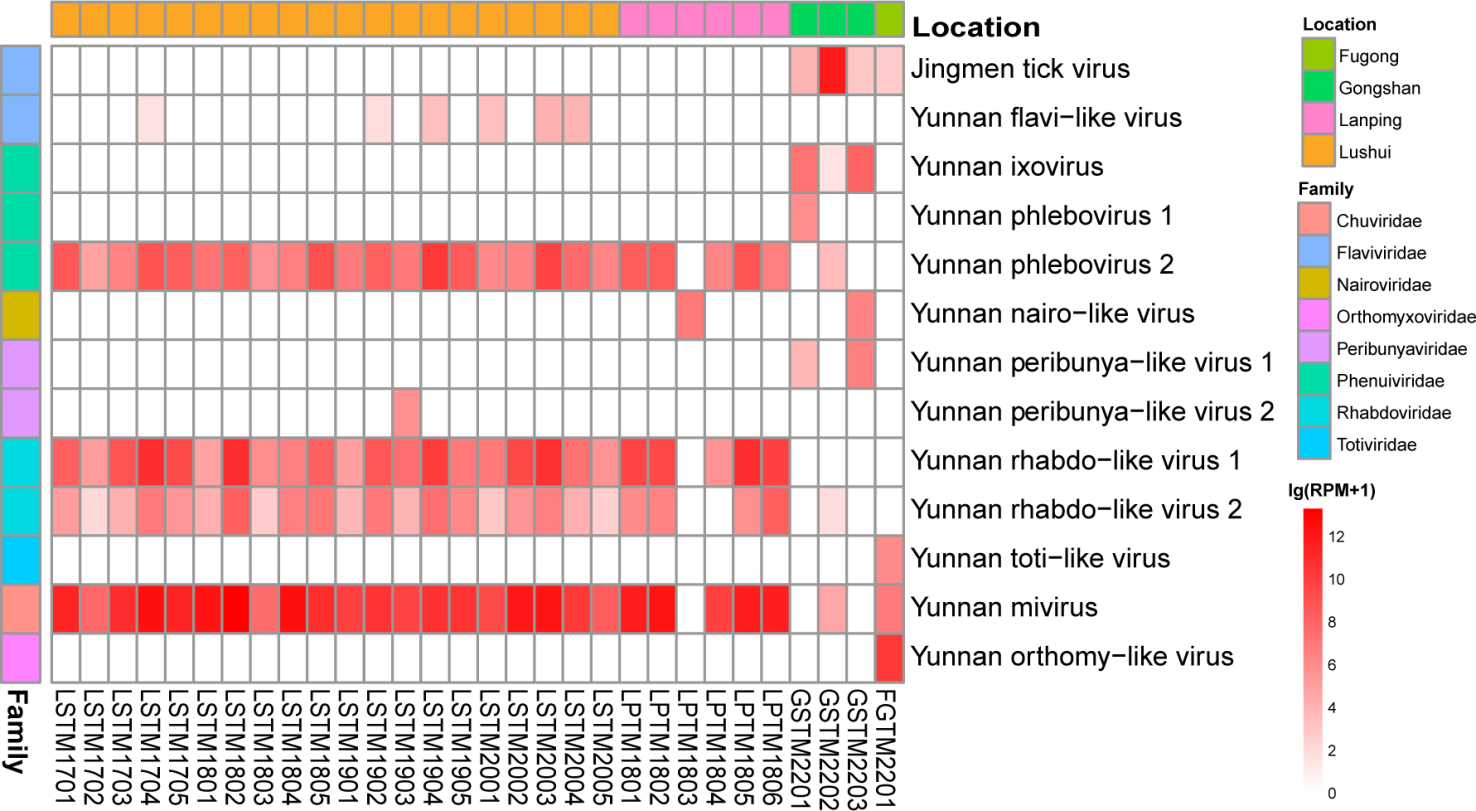
Heatmap showing the prevalence and abundance of viruses identified here (measured by RPM) by meta-transcriptomic sequencing in 30 libraries.

To estimate the distribution and abundance of these viruses across the different libraries, we calculated the reads per million (RPM) for each library and showed the results on a heat map. A higher abundance of YPV2, YRV1, YRV2, and YMV in most libraries reveals they are commonly infected by ticks in Nujiang. Notably, viruses such as YIV, YPBV1, YRV1, YOV, and YTV exhibit unique regional endemism, with a primary distribution in the FG and GS. Interestingly, despite lower virus abundance observed in FG and GS relative to LS and LP, the viral diversity was marked higher in FG and GS (**Figure 2**, **Table S3**).

### 3.3 Viral diversity and phylogeny of potential arboviruses

**Figure 3:**
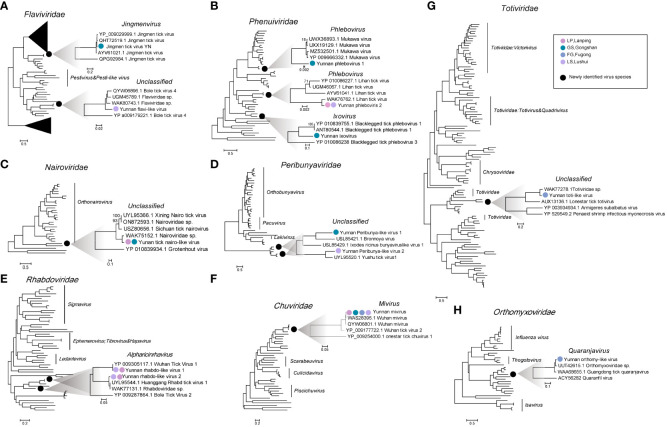
The diversity and evolutionary relationships of the viruses obtained in this study. Eight phylogenetic trees were constructed. Seven of them were based on the RdRp amino acid sequences **(B-H)**, and one on the nonstructural protein NS5 of Flaviviridae **(A)**. All show the positions of the newly discovered virus in the respective virus families. Black circles indicate the currently obtained virus species in each virus family; circles of different colors indicate the corresponding sampling sites on the right of the evolutionary tree. The legend is at the top right.

Based on the RdRp protein of the newly discovered viruses, the phylogenetic analysis aligned with those related viral reference sequences downloaded from the National Center for Biotechnology Information (NCBI) showed that these 13 viruses were found within the trees of eight known viral families. Of note, most of these viruses were either unclassified genera or species (**Figure 3**), with the most remarkable diversity found in the *Phenuiviridae* (n = 3), *Peribunyaviridae* (n = 2), and *Flaviviridae* (n = 2) (**Figure 3**, **Table 1**).

#### 3.3.1 *Flaviviridae*: Jingmen tick virus

**Figure 4:**
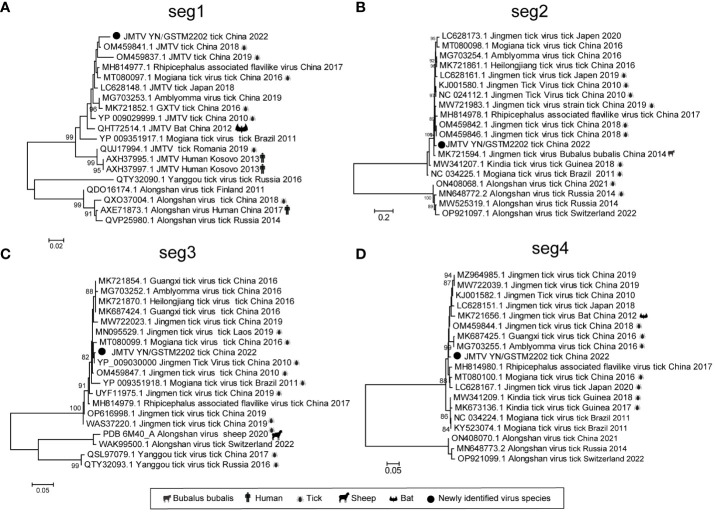
Phylogenetic trees of JMTV based on the NSP1 **(A)**, VP1 **(B)**, NS2B/NS3 **(C)** and VP2/3 **(D)** proteins. Black circles indicate the JMTV in this study. The right icon of the evolutionary tree shows the host of the reference sequence.

The current study obtained the four segments of the Jingmen tick virus (JMTV) from the GS library. Segment 1 (3,121 bp) encoding NSP1, closely resembling the NS5 protein of flaviviruses, segment 3 (2,850 bp) encoding NSP2, is similar to the NS2B/NS3 protein complex of flaviviruses (Qin et al., 2014). Additionally, segment 2 (2,762 bp) encoding VP1 and segment 4 (2,802 bp) encoding VP2/3 protein (**Table 1**). The four segments of the JMTV show a high degree of homology, ranging from 97.35% to 99.12% (aa) with other validated JMTVs (**Table 1**). Our phylogenetic analysis revealed that the JMTV clustered together with other JMTVs. Particularly the NSP1 sequence most closely related to JMTV (OM459841.1), which was found in *Rhipicephalus microplus* from Guizhou in 2018 and closely associated with JMTV strains obtained from humans (AXH37995.1) in Kosovo and bats (QHT72514.1) in China (**Figures 4A–D**).

#### 3.3.2 *Nairoviridae*: Yunnan nairo-like virus

The family *Nairoviridae*, part of the *Bunyavirale*s order, consists of several species, many of which are tick-borne viruses. Some members of this family are known to be pathogenic to mammals, including humans (Lasecka and Baron, 2014). This research identified the L and S segments in the LP and GS libraries. Notably, the L protein of one of the viruses, namely, Yunnan nairo-like virus (YNV), shares 65.31% aa identity with an unclassified *Nairoviridae* virus (USZ80660.1), and the S protein demonstrated low similarity (54.91%) to known viruses (**Table 1**). The phylogenetic tree put the YNV into an unclassified virus clade and formed a distinct branch with a pada-originated virus in Sichuan Province, namely, Sichuan tick nairovirus (USZ80656.1) (**Figure 3C**).

#### 3.3.3 *Phenuiviridae*: Yunnan phlebovirus 1, Yunnan phlebovirus 2, and Yunnan ixovirus

The *Phenuiviridae* family belongs to the *Bunyavirales* order, and its genome consists of three RNA segments: L, M, and S. In this study, we identified three viruses belonging to two viral genera within the *Phenuiviridae* family. In the phylogenetic tree of the RNA-dependent RNA polymerase (RdRp) of the *Phenuiviridae* family, two members were named YPV1 and YPV2 within the Phlebovirus genus. The other, namely, YIV, was clustered into the Ixovirus genus (**Figure 3B**). Genome sequencing revealed that these viruses possess both L and S segments, with the YPV1 having an M segment. The YPV1 genome, derived from the GS library, displayed a high level of identity to the Mukawa virus (MKWV) with 91.80% aa similarity in L protein (WAK75785.1), 85.26% aa similarity in M protein (YP_009666331.1), and 85.54% aa similarity in partial S protein (**Table 1**). In the RdRp phylogenetic tree, YPV1 clustered in the same clade with the Mukawa virus identified in China and Japan (**Figure 3B**, **Table 1**). A separate Phlebovirus, named Yunnan phlebovirus 2 (YPV2), identified as the Lihan virus (LHV), exhibited 99.81% aa identity with the L protein encoding RdRp of other phleboviruses (QCB64646.1) (**Table 1**). The phylogenetic tree shows that our derived LHV clustered with LHVs from various regions (**Figure 3B**). In addition, interestingly, we assembled extremely similar YPV2 sequences from 23 libraries across LP and LS, suggesting that LHV exists widely distributed in Nujiang. In addition to the existing phlebovirus species, we also identified another virus of the phlebovirus from the library of GS, belonging to the Ixovirus, and named YIV. YIV’s L and S segments were 6,740 bp and 2,502 bp, respectively, and shared a 61.23% identity with the RdRp of blacklegged tick phlebovirus 2, first identified in the USA in 2008 (Tokarz et al., 2014) (**Table 1**). The phylogenetic tree showed that the YIV sequence formed an independent evolutionary clade within the Ixovirus genus (**Figure 3B**), indicating that the YIV obtained here could be a novel species of the Ixovirus genus.

#### 3.3.4 *Peribunyaviridae*: Yunnan peribunya-like virus 1 and Yunnan peribunya-like virus 2

In the current study, we identified two unclassified Peribunya viruses in the LS and GS libraries, tentatively named YPBV1 and YPBV2. The genetic evolutionary analysis showed that YPBV1 and YPBV2 belong to the unclassified Peribunya viruses group but are clustered into two distinct branches (**Figure 3D**). In addition, YPBV1 shared only 62.71% aa similarity with the L segment of the Bronnoya virus (WCD38987.1), whereas YPBV2 showed 79.7% aa homology with Yushu tick virus 1 (UYL95520.1). These findings suggest that the newly discovered viruses could be novel species within the family *Peribunyaviridae* (**Table 1**).

## 4 Discussion

Ticks, transient blood-sucking parasites residing on the body surface of vertebrates, can transmit a range of pathogens such as viruses, bacteria, and parasites while feeding on various hosts. As crucial arthropod vectors of disease, its epidemiological importance is secondary to the mosquito (Dantas-Torres et al., 2012). Tick bites can cause co-infections with multiple pathogens, leading to the worsening of illness, complications, and prolonging of the disease course, increasing the disease burden in populations (Boyer et al., 2022). Ticks, constituents of the order *Ixodida*, family *Ixodoidea*, number over 900 species worldwide, primarily from the *Argasidae*, *Ixodida*e, and *Nuttalliellidae* (Horak et al., 2002). In China, 125 tick species from nine genera, including 111 hard tick species and 14 soft tick species, have been reported, and many are important vectors in transmitting zoonoses (Zhang et al., 2019).

With the application of high-throughput sequencing technologies in the last 15 years, an array of emerging tick-borne viruses has been identified, thereby expanding the range and diversity of tick-borne viruses (Harvey et al., 2019; Xu et al., 2021; Ortiz-Baez et al., 2023). There has been a progressive focus on their pathogenicity, especially regarding those causing severe illnesses, such as the Tacheng tick virus 2 (TcTV2) of bunyaviruses and the Alongshan virus of flaviviruses (Zhou et al., 2023). In this study, we applied meta-transcriptomic analyses to investigate the diversity of tick-borne virome in Nujiang, identified four distinct species of ticks and obtained 13 different viruses. These data indicate the extremely high genetic diversity and abundance of viruses, including eight validated species and five novel species. *Bunyavirales* was the most abundant, containing six viruses from three families. The present study also classified the genetic evolutionary relationship among Jingmen tick virus (JMTV), YPV1, and YPV2. Significantly, most of the viruses identified here are closely related to other arthropods and many exhibit high prevalence and abundance across tick populations (e.g., YPV2/YRV1/YRV2/YMV), suggesting that these are probably tick viruses as opposed to livestock-associated viruses.

The current study identified four tick species (*R. microplus*, *R. haemaphysaloides*, *H. longicornis*, and *I. ovatus*) belonging to three genera by analyzing the assembled COI genes. *R. microplus* is the dominant tick species distributed and endemic throughout Nujiang. Whether other tick species will become prevalent within Nujiang due to human activity warrants continued surveillance. Several studies propose potential connections tick species may be related to viral groups (Xu et al., 2021). Unfortunately, we encountered issues of varying results between morphological and molecular species identification approaches, complicating tick species representation in the library and thus hampering the analysis of the biological correlation between the viral species and their hosts.

The *Flaviviridae* family comprises the genera *Orthoflavivirus*, *Pestivirus*, *Pegivirus*, and *Hepacivirus* and also encompasses some unclassified viruses, belong to the single-stranded positive-stranded RNA viruses (Neufeldt et al., 2018; ICTV, 2023). The Jingmen tick virus (JMTV) was first reported in Jingmen, Hubei Province, from *Rhipicephalus microplus*, which was an unclassified, segmented RNA virus of the *Flaviviridae* family by the ICTV (Qin et al., 2014). Its genome comprises four segments, two of which are associated with the flavivirus structural proteins NS2B/VS3 and NS5 proteins (Qin et al., 2014). JMTV has been reported with high prevalence in *R. microplus* worldwide (Colmant et al., 2022). For instance, JMTV was found in eight tick species in Hubei, with a prevalence of 63% in *R. microplus* and 55% in *H. longicornis* (Qin et al., 2014). The detection rates of JMTV in *R. microplus* and *H. hystricis* reached 53.1% and 46.1% in Zhejiang, China (Qin et al., 2014; Guo et al., 2020). Increasingly, growing evidence proved that JMTV and Alongshan virus (ALSV) can cause symptoms including fever and headache in humans, suggesting that JMTV is transmitted not only exclusively in ticks but also horizontally among arthropods, mammals, and humans (Emmerich et al., 2018; Jia et al., 2019; Wang et al., 2019). The presence of JMTV in GS threatens local people’s livestock economy and health; therefore, it is necessary to strengthen the continuous monitoring and control of local tick species and JMTV.

This study also identified six bunyaviruses distributed among three families (*Phenuiviridae*, *Peribunyaviridae*, and *Nairoviridae*). Most of them are unclassified, except YPV1 and YPV2. In recent years, several human and livestock febrile illnesses have been caused by tick-borne bunyaviruses, and most patients have symptoms such as headache, fever, depression, fatigue, and dizziness (Ma et al., 2021; Wang et al., 2021b). Other researchers have identified a variety of viruses from the sera of people bitten by ticks, including the significant tick-transmitted pathogens Crimean-Congo hemorrhagic fever virus (CCHFV) and Nairobi sheep disease virus (NSDv) (Lasecka and Baron, 2014). CCHFV can cause hemorrhagic fever in humans with a mortality rate of up to 30% (Kong et al., 2022); NSDv can cause fatal acute hemorrhagic gastroenteritis with a mortality rate of more than 90% in mammals in Africa and India (Marczinke and Nichol, 2002). The phylogenetic tree showed a close link between the identified bunyaviruses and ticks or other arthropods, indicating possible spread between ticks; further studies are needed to confirm potential risks of spillover into humans (**Figures 3B–D**). Among them, YPV2 was highly prevalent in 23 libraries in NJ within the family *Phenuiviridae*. It presented low host specificity and was considered as a tick endosymbionts (Li et al., 2015a; Temmam et al., 2019). In addition, a virus similar to Mukawa virus called YPV1 was identified in GS. Mukawa virus is a novel tick-borne virus in the genus Phlebovirus of the family *Phenuiviridae*, first reported in *Ixodes persulcatus* in Japan, which was revealed capable of replicating in mammalian cells and potentially causing human infections (Matsuno et al., 2018; Wang et al., 2022). Since the potential pathogenicity of these bunyaviruses, further research is needed to investigate their epidemiology and pathogenesis, encompassing their distribution, virulence, and interactions with the host.

There are several limitations in this study. Firstly, inexperience with tick morphology led to co-mingling of various tick species in a single library, preventing the study of interdependence and transmission relationships between pathogens and host species. Second, the low sampling frequency and the small number of samples at some sampling sites lead to the results not adequately revealing complete virome within the region. Additionally, because of pooling of the samples, there are possibilities of missing out on some low-abundance pathogens. The potential pathogenicity of the newly identified viruses toward humans and other mammals remains unclear, and further studies such as tissue culture and serological tests are warranted. Consequently, future efforts will evaluate pathogenicity in select tick-borne viruses identified in this study. We will expand the collection range and sample size to better characterize the distribution of ticks and the prevalence of tick-borne viruses in Nujiang, which provides the necessary background information for preventive measures against tick-borne diseases.

## Data availability statement

The data presented in the study are deposited in the GenBank repository, accession number from OR892588 to OR892614.

## Ethics statement

The study protocol was approved by the Medical Ethics Committee of the Yunnan Institute of Endemic Diseases Control and Prevention (File No. 20210016).

## Author contributions

JuW: Methodology, Software, Visualization, Writing – review & editing. JiW: Methodology, Software, Writing – review & editing. GK: Investigation, Methodology, Writing – review & editing. WW: Supervision, Writing – review & editing. LY: Investigation, Methodology, Writing – review & editing. WY: Investigation, Writing – review & editing. HP: Investigation, Writing – review & editing. XH: Investigation, Methodology, Writing – review & editing. TY: Investigation, Methodology, Validation, Visualization, Writing – review & editing. MS: Data curation, Investigation, Methodology, Project administration, Supervision, Writing – review & editing. YF: Investigation, Writing – review & editing.
